# Exploring the In Vitro Mechanism of Action of β-Acetoxyisovalerylalkannin on Inflammatory Skin Diseases Using Network-Based Pharmacology and Non-Targeted Metabolomics

**DOI:** 10.3390/ph18091249

**Published:** 2025-08-22

**Authors:** Yinglan Ma, Xuehong Ma, Yue Ma, Liuqian Peng, Zixin Zhang, Jinyan Li, Lu Zhang, Jianguang Li

**Affiliations:** 1 College of Pharmacy, Xinjiang Medical University, Urumqi 830011, China; myl2443382113@163.com (Y.M.); mxh6867@163.com (X.M.); mayue9132@163.com (Y.M.); 13150350230@163.com (L.P.); zhangzixinkk@163.com (Z.Z.); jinyanli1015@163.com (J.L.); 2 Xinjiang Key Laboratory of Natural Medicines Active Components and Drug Release Technology, Xinjiang Medical University, Urumqi 830011, China; 3 Xinjiang Key Laboratory of Biopharmaceuticals and Medical Devices, Xinjiang Medical University, Urumqi 830011, China; 4 Science and Technology Innovation and Transformation Service Center, Xinjiang Medical University, Urumqi 830011, China; 5 College of Pharmacy, Xinjiang Second Medical College, Karamay 834000, China

**Keywords:** skin inflammation, β-Acetoxyisovalerylalkannin, network pharmacology, untargeted metabolomics

## Abstract

**Objective:** Lithospermum erythrorhizon has been extensively used for the clinical treatment of skin diseases, but its material basis and mechanism of action remain unclear. This study integrates network pharmacology, untargeted metabolomics, and in vitro experimental validation to elucidate the anti-inflammatory effects and underlying mechanisms of β-acetoxyisovalerylalkannin, a bioactive naphthoquinone compound isolated from Arnebiae Radix, using inflammatory skin disease models. **Methods**: Core targets for β-Acetoxyisovalerylalkannin and skin inflammation were identified via network pharmacology and validated through molecular docking. In vitro assays assessed β-Acetoxyisovalerylalkannin’s impact on keratinocyte proliferation, migration, apoptosis, and inflammatory factors (CXCL1, CXCL2, CXCL8, CCL20, IFN-γ, MCP-1, TNF-α, NF-κB). Non-targeted metabolomics identified differential metabolites and pathways. **Results**: Network pharmacology revealed 66 common targets significantly enriched in the MAPK/STAT3 signaling pathway. In vitro, β-Acetoxyisovalerylalkannin suppressed proliferative viability and hypermigration and induced apoptosis in HaCaTs. Moreover, it downregulated the mRNA levels of inflammatory markers (CXCL1, CXCL2, CXCL8, CCL20, IFN-γ, MCP-1, TNF-α, and NF-κB) by inhibiting the activation of the MAPK/STAT3 signaling pathway. Metabolomics identified 177 modified metabolites, associating them with the arginine/proline, glycine/serine/threonine, glutathione, and nitrogen metabolic pathways. **Conclusions**: β-Acetoxyisovalerylalkannin exerts protective effects against skin inflammation by reducing abnormal cell proliferation and inflammatory responses, promoting apoptosis, and effectively improving the metabolic abnormalities of HaCaTs. β-Acetoxyisovalerylalkannin is, therefore, a potential therapeutic option for mitigating skin inflammation-related damage.

## 1. Introduction

Inflammatory skin diseases are a diverse group of disorders characterized by immune system dysregulation, leading to the destruction of skin tissue. Common conditions within this category include psoriasis, atopic dermatitis, eczema, vitiligo, and hives. With environmental changes and evolving lifestyles, the incidence of these diseases continues to increase globally. Millions of individuals suffer from these diseases, which are often marked by severe disruption of the skin barrier and chronic inflammation [1]. The pathogenesis of inflammatory skin diseases is multifactorial and not fully understood; these conditions are typically triggered by both genetic and environmental factors, and they are immune-mediated, chronic, relapsing, and systemic in nature, making them difficult to treat and requiring long-term management [2,3]. Modern therapies mainly rely on using hormones and immune-suppressing drugs to manage symptoms, offering some relief for patients. Unfortunately, long-term or incorrect usage frequently leads to severe adverse reactions and toxicity. Furthermore, their long treatment durations and high economic costs make these therapies unsuitable for long-term use.

Arnebiae Radix, a staple in traditional Chinese medicine, has various healing benefits. This potent herb is renowned for promoting tissue repair, combating infections, and reducing inflammation. It also excels at purging toxins, dispersing heat, improving blood flow, and soothing skin irritations. With its multifaceted therapeutic properties, it can treat conditions ranging from internal imbalances to dermatological issues [4]. With a long history of external use, Arnebiae Radix has proven effective in treating numerous skin disorders, gynecological conditions, and wound ulcers [5]. The medicinal properties of Arnebiae Radix are largely attributed to its rich array of active ingredients, including naphthoquinones, polysaccharides, volatile oils, and organic acids [6]. Previous studies have highlighted the therapeutic potential of Arnebiae Radix’s naphthoquinone compounds, such as leucovorin, deoxynivalenol, and acetylakannin [7]. While much research has focused on leucovorin, systematic studies on other naphthoquinone-rich compounds in comfrey, which also exhibit anti-inflammatory activities, remain limited.

β-Acetoxyisovalerylalkannin, a naphthoquinone-rich compound found in Arnebiae Radix, is primarily sourced from the dried roots of plants in the Arnebiae Radixaceae family. This compound has remarkable therapeutic potential, demonstrating a broad spectrum of pharmacological activities, including antitumor [8], anti-inflammatory [9], antimicrobial [10], and antiviral [11] effects. Moreover, it is useful for treating toxicity, having a negligible impact on human health and very few side effects [12]. Human immortalized keratinocytes (HaCaT) are often used as models for studying pathological changes associated with inflammatory skin diseases. In response to inflammation-related stimuli, keratinocytes activate and release varied downstream inflammatory factors, maintaining localized inflammation in the skin. This research investigates how β-Acetoxyisovalerylalkannin suppresses inflammation in HaCaT cells through a combination of network pharmacology and untargeted metabolomics. By analyzing these molecular interactions, we establish a scientific foundation for its therapeutic application in managing inflammatory skin conditions. This study sheds light on both the compound’s inhibitory properties and its mode of action at the cellular level.

## 2. Results

### 2.1. Network Pharmacology and Molecular Docking Analysis of β-Acetoxyisovalerylalkannin

**Figure 1:**
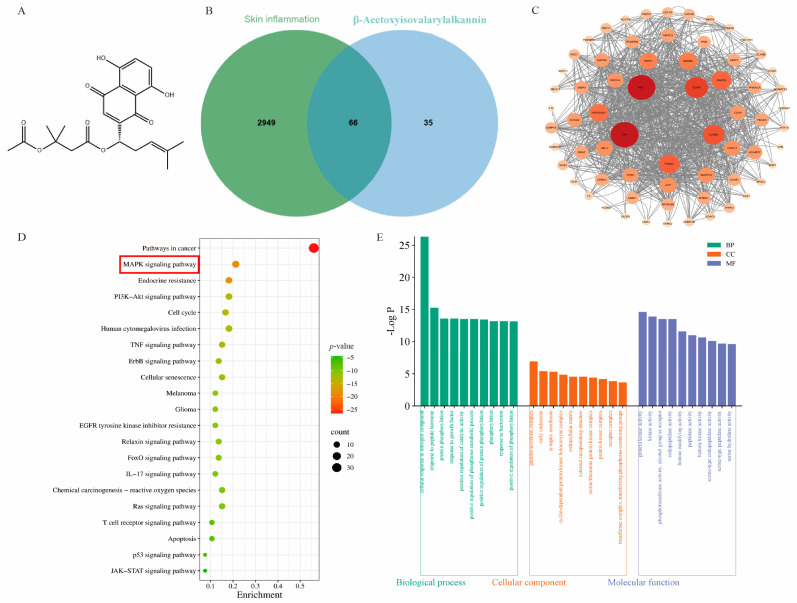

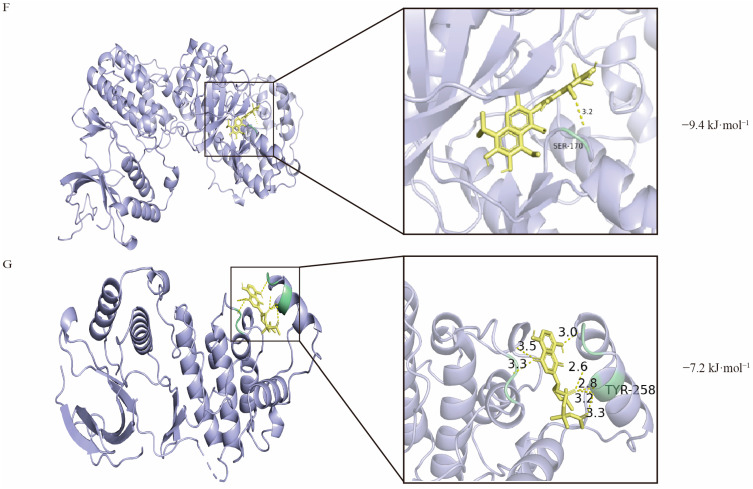
Network pharmacology and molecular docking. (**A**) β-Acetoxyisovalarylalkannin structural formula; (**B**) Venn diagram; (**C**) protein interaction network diagram (PPI diagram); (**D**,**E**) KEGG path enrichment analysis and GO diagram; (**F**,**G**) molecular docking results of β-Acetoxyisovalerylalkannin with ERK1/2 and p38 (Blue represents the protein structure, yellow represents the drug molecule structure, and green represents the amino acid residue).

To elucidate the potential therapeutic mechanisms of β-Acetoxyisovalerylalkannin in skin inflammation, we conducted an integrated network pharmacology and molecular docking study. Our analysis identified 66 overlapping targets between β-Acetoxyisovalerylalkannin and inflammatory skin diseases, as demonstrated via Venn diagram analysis (Figure 1B). The study objectives were then pursued through constructing a protein–protein interaction network and performing a functional treasure hunt. An analysis of the KEGG pathways showed that the anti-inflammatory prowess of β-Acetoxyisovalerylalkannin largely depended on adapting four key signaling channels: the MAPK, PI3K/AKT, TNF, and JAK-STAT pathways. Initially, molecular docking was used to check the compatibility between β-Acetoxyisovalerylalkannin and the two main elements in the MAPK pathway: ERK1 and p38. The binding energies of −9.4 kJ·mol^−1^ for ERK1 and −7.2 kJ·mol^−1^ for p38 indicated strong ligand–receptor interactions, with negative values suggesting favorable binding affinity. These findings suggest that β-Acetoxyisovalerylalkannin may exert its anti-inflammatory effects through the targeted regulation of the MAPK signaling pathway. The robust binding interactions observed with ERK1 and p38 proteins provide molecular-level evidence supporting the compound’s potential as a therapeutic agent for treating inflammatory skin conditions.

### 2.2. Effect of β-Acetoxyisovalerylalkannin on HaCaT Cell Proliferation

**Figure 2:**
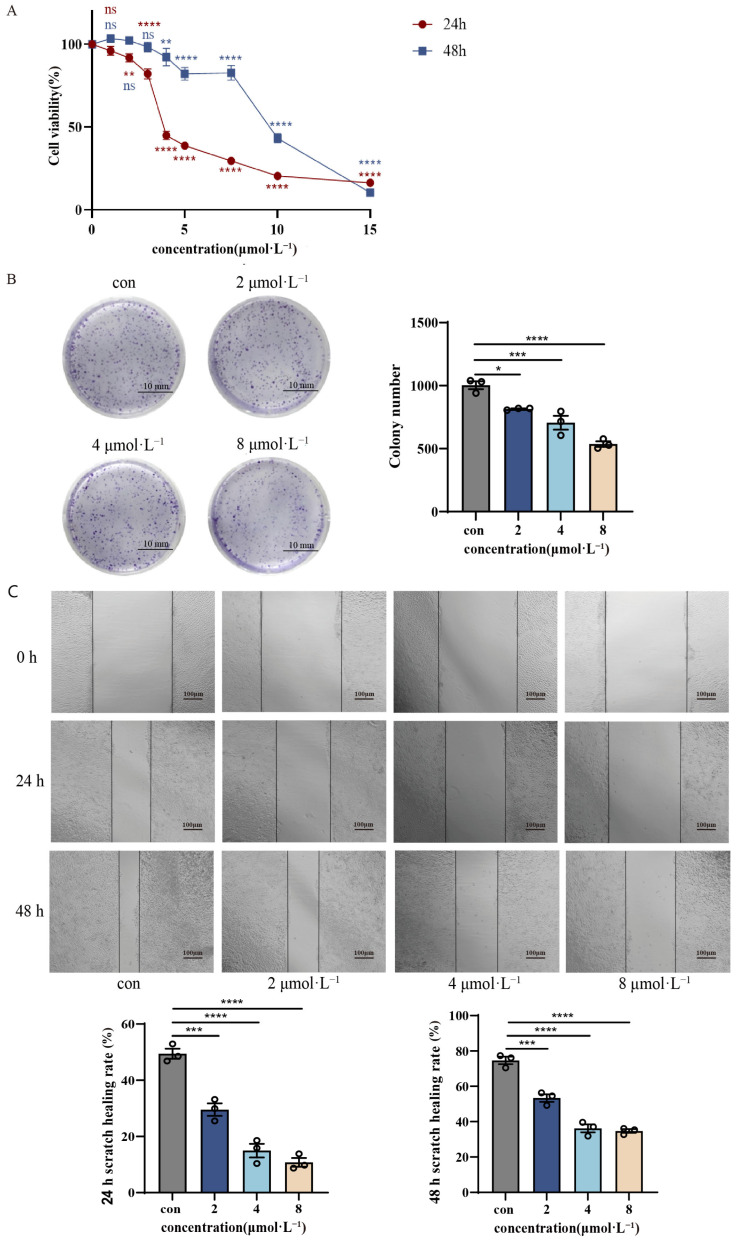
The effect of β-Acetoxyisovalerylalkannin on the proliferation and migration of HaCaT cells: (**A**) CCK-8 growth curves of β-Acetoxyisovalerylalkannin-treated HaCaT cells; (**B**) typical images and quantification of plate clones of HaCaT cells treated with β-Acetoxyisovalerylalkannin (scale bar, 10 mm); (**C**) typical images and quantification of the scratch wound healing of HaCaT cells treated with β-Acetoxyisovalerylalkannin (n = 3, scale bar, 100 μm). Data are mean ± SD. Statistical significance is: ns, indicating no significant difference; * *p* < 0.05, ** *p* < 0.01, *** *p* < 0.001, and **** *p* < 0.0001 vs. con, n = 3, analyzed via one-way ANOVA.

HaCaT cells were exposed to β-Acetoxyisovalarylalkannin at concentrations ranging from 0 to 15 μmol·L^−1^. Our findings demonstrated that cell viability decreases significantly with increasing concentrations of β-Acetoxyisovalerylalkannin administration (Figure 2A). The half-maximal inhibitory concentration (IC_50_) of β-Acetoxyisovalerylalkannin in HaCaT cells was 4.6 μmol·L^−1^, demonstrating that β-Acetoxyisovalerylalkannin significantly inhibits HaCaT cell proliferation within the concentration range of 2–15 μmol·L^−1^, exhibiting potent dose-dependent anti-proliferative activity. Colony formation assays revealed a significant, dose-dependent decrease in HaCaT cell clonogenic capacity following 48 h of treatment with β-Acetoxyisovalerylalkannin compared to untreated controls (Figure 2B). These data collectively indicate that β-Acetoxyisovalerylalkannin effectively suppresses HaCaT cell proliferation by impairing colony-forming ability. Cell migration capacity was determined through scratch wound healing tests. Treatment groups (low, medium, and high doses) exhibited markedly reduced wound closure rates compared to the control, with migration inhibition showing concentration-dependent characteristics (Figure 2C). Statistical analysis confirmed the significance of these observations (*p* < 0.001), demonstrating that β-Acetoxyisovalerylalkannin potently inhibits HaCaT cell migration in vitro.

### 2.3. Impact of β-Acetoxyisovalerylalkannin-Induced Apoptosis on HaCaTs

**Figure 3:**
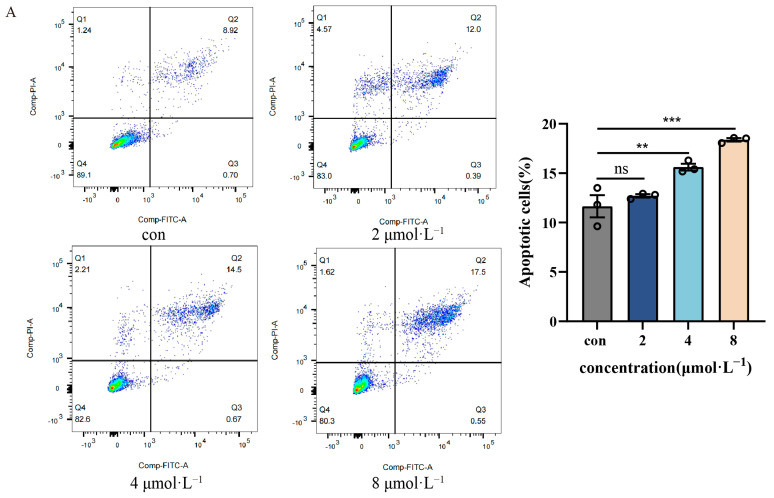

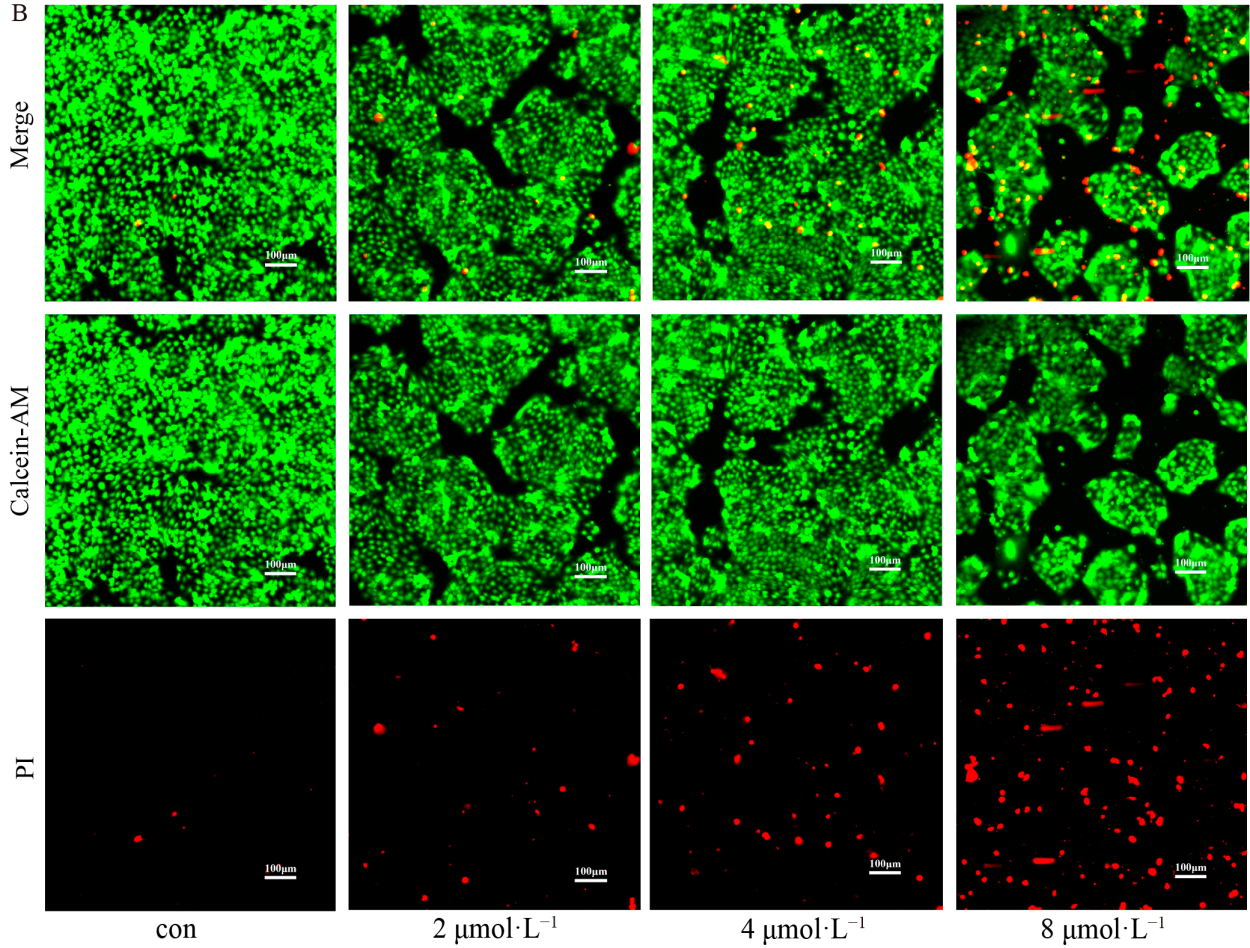
The effect of β-Acetoxyisovalerylalkannin on the apoptosis of HaCaT cells: (**A**) flow cytometry was used to analyze the apoptosis of HaCaT cells after treatment with β-Acetoxyisovalerylalkannin; (**B**) the detection of apoptosis via immunofluorescence (green for normal viable cells, red for apoptotic cells; n = 3, scale bar, 100 μm). Data are mean ± SD. Statistical significance is: ns, indicating no significant difference; ** *p* < 0.01 and *** *p* < 0.001 vs. con, n = 3, analyzed via one-way ANOVA.

β-Acetoxyisovalerylalkannin’s pro-apoptotic impact on HaCaT cells was evaluated quantitatively using annexin V-FITC/propidium iodide (PI) flow cytometry. Treatment with increasing concentrations of β-Acetoxyisovalerylalkannin induced dose-dependent increases in both early (Q2 quadrant) and late (Q3 quadrant) apoptotic cell populations (Figure 3A). Statistical analysis revealed a significant increase in total apoptosis rates in the mid- (*p* < 0.01) and high-dose (*p* < 0.001) groups compared to the vehicle-treated controls, confirming the compound’s apoptosis-inducing activity. Complementary immunofluorescence staining using Calcein-AM/PI confirmed these findings, demonstrating a progressive reduction in viable (Calcein-AM) cells and a concomitant increase in apoptotic (PI) cells across treatment groups (Figure 3B). This dose-responsive apoptotic effect was observed even at lower concentrations, supporting the pro-apoptotic activity of β-Acetoxyisovalerylalkannin in human keratinocytes.

### 2.4. Effect of β-Acetoxyisovalerylalkannin on Inflammatory Factors

**Figure 4:**
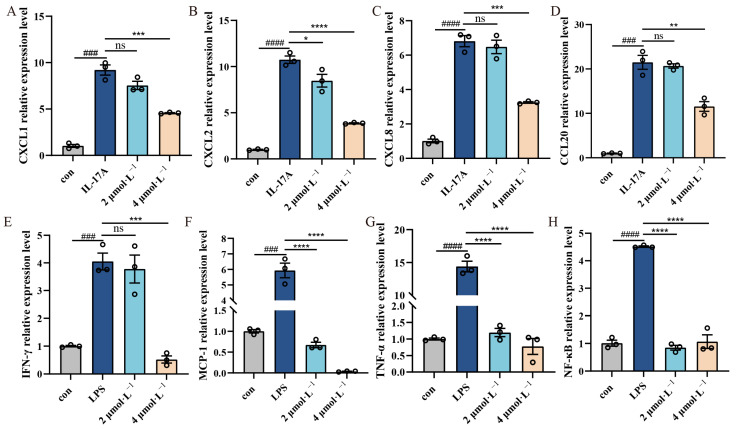
The effect of β-Acetoxyisovalerylalkannin on the expression of related cytokines in HaCaT cells. (**A**–**D**) RT-qPCR was used to detect the mRNA levels of CXCL1, CXCL2, CXCL8, and CCL20 after IL-17A induction; (**E**–**H**) RT-qPCR was used to detect the mRNA levels of IFN-γ, MCP-1, TNF-α, and NF-κB after LPS induction. Data are mean ± SD. ### *p* < 0.001 and #### *p* < 0.0001 vs. con; statistical significance is: ns, indicating no significant difference; * *p* < 0.05, ** *p* < 0.01, *** *p* < 0.001, and **** *p* < 0.0001 vs. IL-17A/LPS, n = 3, analyzed via one-way ANOVA.

Quantitative RT-PCR analysis revealed significant upregulation of inflammatory mediators in stimulated HaCaT cells, with the model groups showing markedly elevated mRNA expression of chemokines (CXCL1, CXCL2, CXCL8, CCL20), proinflammatory cytokines (IFN-γ, MCP-1, TNF-α), and the transcription factor NF-κB (*p* < 0.001). β-Acetoxyisovalerylalkannin treatment demonstrated dose-dependent anti-inflammatory effects: In the IL-17A-induced model, significant suppression of chemokine expression (CXCL1, CXCL2, CXCL8, CCL20) was noted, while the high-dose treatment showed greater efficacy (*p* < 0.01). In contrast, in the LPS-induced model, potent inhibition of proinflammatory cytokines (IFN-γ, MCP-1, TNF-α) and NF-κB was noted, with both low- and high-dose treatments being effective (*p* < 0.001). These results suggest that β-Acetoxyisovalerylalkannin modulates multiple inflammatory pathways in keratinocytes (Figure 4).

### 2.5. β-Acetoxyisovalerylalkannin-Inhibited MAPK/STAT3 Signaling Pathway

**Figure 5:**
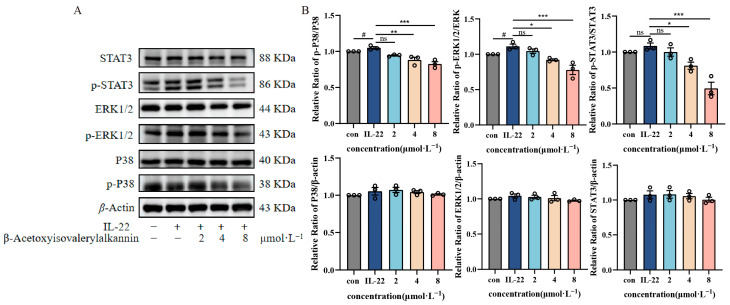
β-Acetoxyisovalerylalkannin activates the intracellular MAPK/STAT3 signaling pathway: (**A**) protein expression of p-P38, P38, p-ERK1/2, ERK1/2, p-STAT3, and STAT3 in HaCaT treated with β-Acetoxyisovalerylalkannin; (**B**) quantification of the protein expression of p-P38, P38, p-ERK1/2, ERK1/2, p-STAT3, and STAT3 (n = 3). Data are mean ± SD. Statistical significance is: ns, indicating no significant difference; # *p* < 0.05 vs. con; statistical significance is ns, indicating no significant difference; * *p* < 0.05, ** *p* < 0.01, and *** *p* < 0.001 vs. IL-22, n = 3; analyzed via one-way ANOVA.

The MAPK/STAT3 pathway is crucial for modulating cellular functions such as inflammation, growth, and programmed cell death. To examine the effects of β-Acetoxyisovalerylalkannin on this signaling cascade, we analyzed the expression and activation states of critical proteins—P38, ERK1/2, and STAT3—along with their phosphorylated counterparts (p-P38, p-ERK1/2, p-STAT3) using Western blot analysis in HaCaT cells. In this study, β-Acetoxyisovalerylalkannin had differential inhibitory effects on MAPK/STAT3 signaling pathway components depending on dosage concentrations. While β-Acetoxyisovalerylalkannin treatment did not significantly alter the total protein expression levels of P38, ERK1/2, and STAT3, we observed concentration-dependent reductions in their phosphorylated forms (p-P38, p-ERK1/2, and p-STAT3). Comparative analysis with the model group revealed that the low-dose group (2 μmol·L^−1^) failed to effectively suppress the expression of phosphorylated proteins. However, both medium- (4 μmol·L^−1^) and high-dose (8 μmol·L^−1^) groups demonstrated significant inhibition of p-P38, p-ERK1/2, and p-STAT3 protein expression, with the most pronounced effects being observed in the high-dose group (Figure 5). These findings suggest that β-Acetoxyisovalerylalkannin exerts its anti-inflammatory activity by modulating the aberrant activation of the MAPK/STAT3 signaling pathway within a specific concentration range (4–8 μmol·L^−1^).

### 2.6. Non-Targeted Metabolomics Analysis

**Figure 6:**
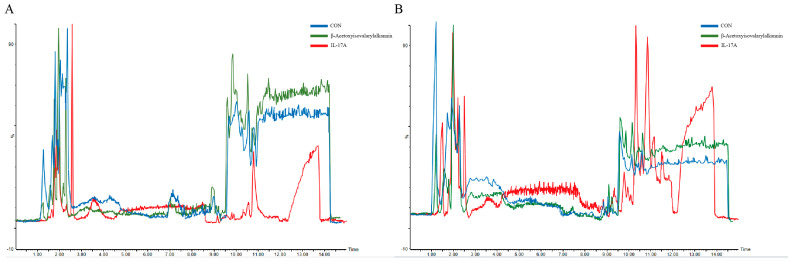

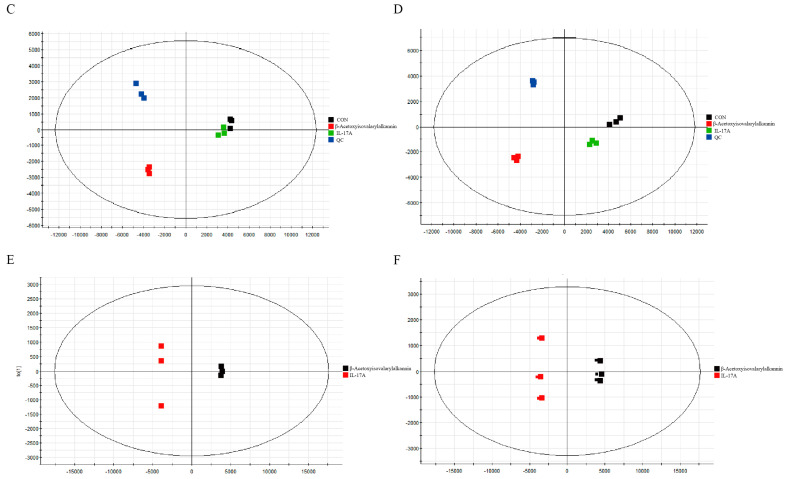
(**A**,**B**) Ion plots for positive and negative ion modes; (**C**,**D**) PCA score plots for positive and negative patterns; (**E**,**F**) OPLS–DA plots in positive and negative modes.

Comprehensive metabolomic investigations were conducted with UPLC-Q-TOF/MS, employing both positive and negative ESI settings. Total ion chromatograms (TICs) demonstrated distinct peak profiles and intensity distributions among the control, model (IL-17A-treated), and β-Acetoxyisovalerylalkannin-treated groups. Unsupervised principal component analysis (PCA) of quality control (QC) samples exhibited tight clustering in both ionization modes (R^2^ > 0.85), confirming analytical method reliability and reproducibility. Biological replicates showed excellent intra-group clustering with clear inter-group separation in PCA score plots, indicating significant metabolic differences. Supervised orthogonal partial least squares–discriminant analysis (OPLS–DA) further validated complete separation between model and experimental groups (Q2 > 0.7) and significant metabolic alterations (VIP > 1.5, *p* < 0.05) in experimental versus IL-17A model groups. These results (Figure 6) demonstrate that β-Acetoxyisovalerylalkannin treatment induces substantial metabolic reprogramming in HaCaT cells, effectively modulating the IL-17A-induced metabolic perturbations.

Through analyzing endogenous metabolites in both positive and negative ion modes and utilizing quantitative ion chromatography (QI), we assessed these substances, pinpointing those of significant importance—those with a VIP score greater than 1 and a *p*-value less than 0.05. Positive ion mode yielded 1233 compounds, while negative ion mode yielded 1535 compounds. Through the HMDB database, we pinpointed 97 differential metabolites in positive ion mode and 80 in negative ion mode. Across both ion modes, we identified 177 endogenous differential metabolites, with 129 being upregulated and 48 being downregulated, as detailed in Table 1.

**Figure 7:**
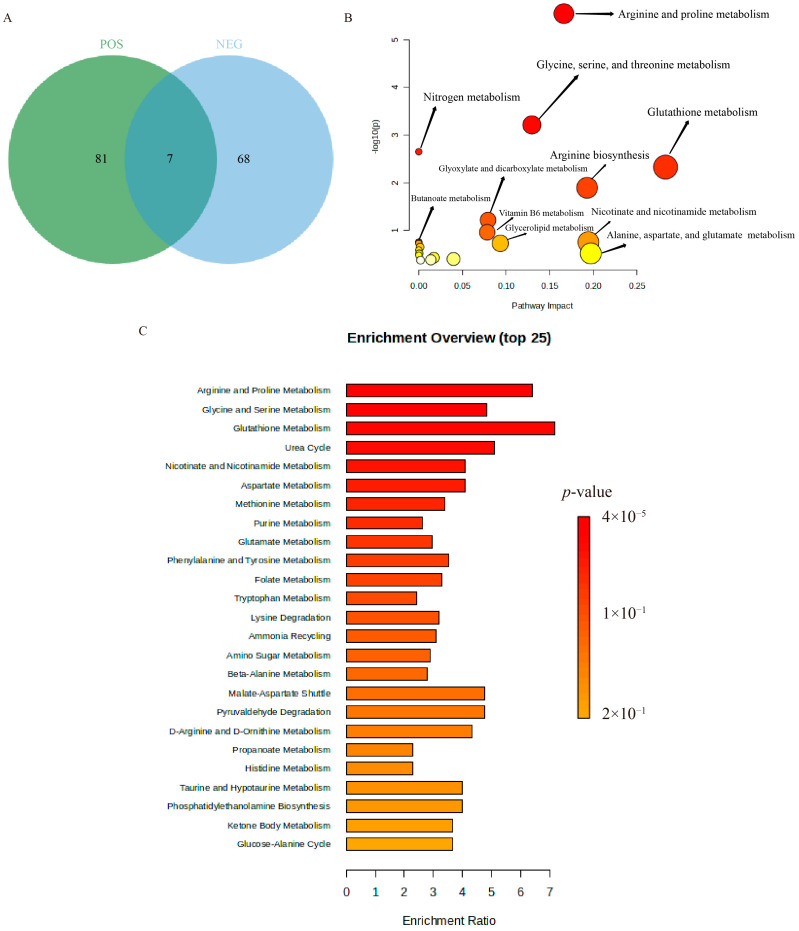
Enrichment analysis plots of different metabolites: (**A**) Venn diagram illustrating overlapping differential metabolites detected in both positive and negative ion modes; (**B**) pathway analysis diagram of differential metabolites; (**C**) KEGG enrichment analysis map.

The overlapping differential metabolites between the two analytical modes were visualized using Venn diagram analysis, revealing seven common endogenous metabolites consistently altered in both positive and negative ion modes (Figure 7A). The identified differential metabolites were then imported into the MetaboAnalyst 5.0 platform for metabolic pathway enrichment analysis. The results indicated that the primary differential metabolic pathways in both the β-Acetoxyisovalerylalkannin and IL-17A model groups included arginine and proline metabolism; glycine, serine, and threonine metabolism; glutathione metabolism; nitrogen metabolism; and arginine biosynthesis (Figure 7B,C).

**Figure 8:**
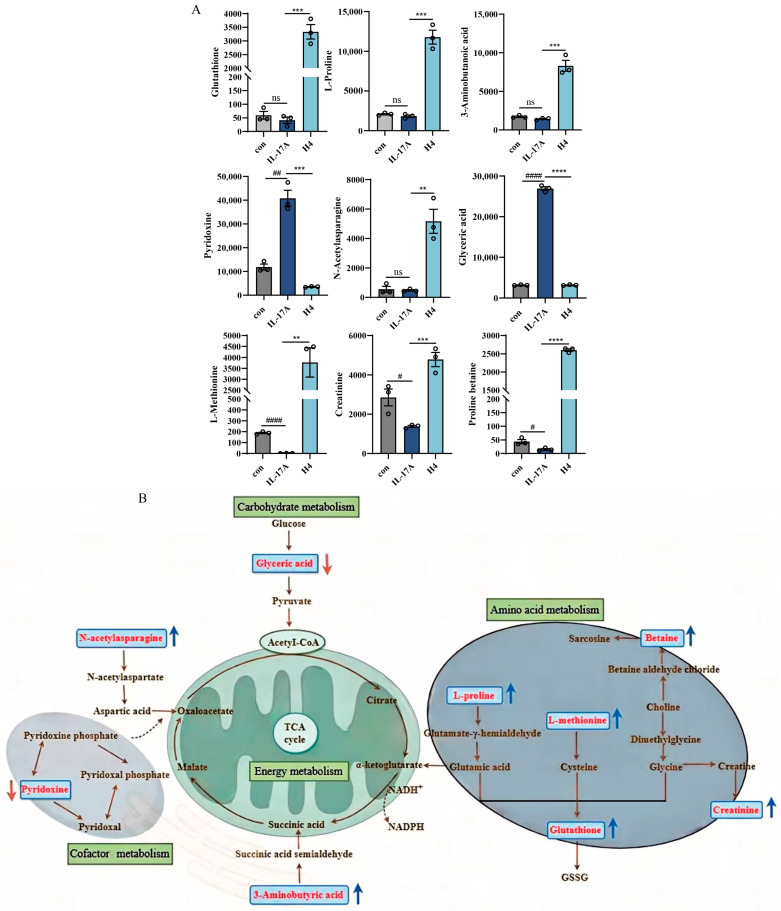
Effect of β-Acetoxyisovalerylalkannin on different metabolites in HaCaT cells: (**A**) changes in differential metabolite levels; (**B**) metabolic pathway diagrams of differential metabolites (red arrows indicate downward adjustments, blue arrows indicate upward adjustments, the dashed arrow indicates an indirect biosynthetic relationship between the two cofactors, the double-headed arrow represents a reversible enzymatic reaction). Data are mean ± SD. Statistical significance is: ns, indicating no significant difference; # *p* < 0.05, ## *p* < 0.01, and #### *p* < 0.0001 vs. con; ** *p* < 0.01, *** *p* < 0.001, and **** *p* < 0.0001 vs. IL-17A, n = 3; analyzed via Student’s *t*-test.

Analysis of key metabolites within the identified metabolic pathways revealed that β-Acetoxyisovalerylalkannin significantly altered the levels of several metabolites in HaCaT cells. Specifically, β-Acetoxyisovalerylalkannin significantly upregulated the levels of glutathione (GSH), L-proline, 3-aminobutyric acid, L-methionine, N-acetyl asparagine, creatinine, and proline betaine. Conversely, it significantly downregulated the levels of glyceric acid and pyridoxine (Figure 8).

## 3. Discussion

Although no cures exist for inflammatory skin diseases, topical, oral, and systemic therapies are available to alleviate symptoms. Research has demonstrated the efficacy of certain plants and phytochemicals for managing inflammatory skin conditions, in both preclinical and clinical studies. These natural compounds are applicable in topical, oral, and systemic treatment modalities. Traditionally, some of these botanicals, including their extracts and/or specific phytochemicals, have served as preferred treatments for individuals with moderate-to-severe psoriasis due to their favorable side effect profiles. This study explored how β-Acetoxyisovalerylalkannin suppresses inflammation in HaCaT cells, using network pharmacology and untargeted metabolomics to analyze its mechanisms. Network pharmacology revealed that β-Acetoxyisovalerylalkannin targets key inflammatory skin disease-associated genes, including MAPK, TNF-α, and AKT. KEGG enrichment analysis indicated that its anti-inflammatory effects primarily involve the MAPK, PI3K/AKT, and JAK/STAT pathways, with the MAPK pathway being key in regulating inflammation and apoptosis mediators during skin inflammation [13]. Molecular docking confirmed high binding affinity to core targets, particularly ERK1/2 and p38 (MAPK pathway components), suggesting that the modulation of the MAPK pathway is a primary anti-inflammatory mechanism.

Arnebiae Radix has long been recognized for its medicinal properties, and its naphthoquinone compounds have been shown to inhibit inflammatory factors such as IL-1β, IL-6, IL-15, IL-23, and IFN-γ, with the overexpression of these inflammatory factors not only disrupting inflammatory homeostasis but also further aggravating the inflammatory response [14]. Studies have shown that shikonin inhibits apoptosis and the expression of inflammatory factors in HaCaT cells in a concentration-dependent manner, as well as promoting cell activity [15,16,17,18,19]. It is noteworthy that while shikonin exhibits potent anti-inflammatory effects in vitro, its free phenolic hydroxyl groups render it susceptible to oxidative degradation [20]. In contrast, β-Acetoxyisovalerylalkannin demonstrates enhanced molecular stability due to the acetylation of its phenolic hydroxyl groups and the consequent increase in steric hindrance. Structural analysis reveals that β-Acetoxyisovalerylalkannin possesses significant advantages over shikonin, exhibiting superior stability under physiological conditions. Our research revealed that β-Acetoxyisovalerylalkannin markedly suppressed the production of critical inflammatory mediators, such as CXCL1, CXCL2, CXCL8, CCL20, IFN-γ, MCP-1, TNF-α, and NF-κB, when cells were exposed to IL-17A and LPS. This study demonstrates that reduced expression levels of MCP-1 and TNF-α significantly attenuate inflammatory responses [21]. Furthermore, oxidative stress and mitochondrial dysfunction were identified as critical contributors to the inflammatory cascade, suggesting their potential interplay in disease pathogenesis [22]. The compound had a clear inhibitory effect on these signaling molecules under inflammatory conditions. These findings suggest that β-Acetoxyisovalerylalkannin can effectively mitigate the inflammatory response in inflammatory skin diseases. Notably, abnormal proliferation and resistance to apoptosis in keratinocytes (KCs) are major contributors to the skin thickening observed in psoriasis [23], and β-Acetoxyisovalerylalkannin not only significantly inhibits HaCaT cell proliferation but also induces apoptosis in these cells.

The MAPK/STAT3 signaling axis controls vital cellular processes, including cell growth, development, genetic activity, and programmed cell death [24]. The inhibition of this pathway has been shown to alleviate symptoms of psoriasis-like dermatitis [25,26], as well as atopic dermatitis [27,28]. In this study, β-Acetoxyisovalerylalkannin treatment significantly reduced the phosphorylation of P38, ERK1/2, and STAT3 in HaCaT cells, aligning with previous studies that demonstrated similar effects on inflammatory pathways [29,30]. This suggests that β-Acetoxyisovalerylalkannin may alleviate skin inflammation by inhibiting the immune response mediated by the MAPK/STAT3 pathway.

Non-targeted metabolomics analysis revealed that β-Acetoxyisovalerylalkannin modulates several important metabolic pathways, including arginine and proline metabolism, glycine–serine–threonine metabolism, glutathione metabolism, and nitrogen metabolism. Glutathione, an essential metabolic modulator boasting antioxidant and anti-inflammatory effects, significantly contributes to immune system maintenance and inflammation regulation [31]. β-Acetoxyisovalerylalkannin upregulates glutathione levels, consistent with the findings of Xiong et al., whose study demonstrated that elevated glutathione levels significantly attenuate TNF-α-induced oxidative stress in HaCaT cells, subsequently suppressing both oxidative stress and inflammatory responses through the NF-κB/MAPK/Nrf2 signaling pathway [32]. Moreover, another study showed that increased glutathione levels help to reduce inflammation and oxidative stress, improving the clinical features of psoriasis [33]. Additionally, pathways related to arginine and proline metabolism are closely associated with psoriasis [34], and β-Acetoxyisovalerylalkannin may exert its effects by modulating these pathways. L-methionine, for example, has been shown to inhibit proinflammatory cytokines [35], and L-proline is known to have protective effects in skin inflammation [36]. Furthermore, 3-aminobutyric acid and glyceric acid are involved in key metabolic processes regulating cell energy metabolism, with decreases in glyceric acid levels potentially alleviating inflammation and abnormal cell differentiation [37,38]. The upregulation of L-glutamate (a GSH precursor) and pyroglutamate (a byproduct of GSH degradation) suggests that β-Acetoxyisovalerylalkannin enhances antioxidant defense mechanisms [39]. β-Acetoxyisovalerylalkannin may suppress NF-κB and MAPK signaling pathways by modulating these metabolic changes, thereby attenuating the production of proinflammatory cytokines (e.g., TNF-α, IL-6). Prostaglandins (PGs), derived from arachidonic acid, serve as crucial regulators of inflammation [40]. The observed upregulation of PG(18:1/18:1) by β-Acetoxyisovalerylalkannin may influence cyclooxygenase (COX) activity, potentially modulating prostaglandin E_2_ (PGE_2_) synthesis and subsequent inflammatory responses. Furthermore, the β-Acetoxyisovalerylalkannin-mediated elevation of cinnamic acid and its derivatives demonstrates its anti-inflammatory properties, likely through COX-2 inhibition and reactive oxygen species (ROS) reduction [41]. Monoglycerides (MGs) and sphingomyelins (SMs) participate in membrane signaling and ceramide biosynthesis. MGs may exert anti-inflammatory effects by suppressing proinflammatory cytokines such as IL-6 and TNF-α. The metabolic products of SMs, including sphingosine-1-phosphate (S1P), can regulate immune cell migration and inflammatory responses through S1P receptors. Notably, the S1P signaling pathway has been shown to inhibit Th17 cell differentiation, thereby attenuating inflammatory responses [42]. The upregulation of MGs and SMs by β-Acetoxyisovalerylalkannin potentially enhances the barrier function of keratinocytes, consequently reducing inflammation triggered by external stimuli [43]. UDP-*N*-acetylglucosamine (UDP-GlcNAc), serving as the essential substrate for protein O-GlcNAcylation, may indirectly suppress inflammation through increased O-GlcNAc modifications that reduce ROS accumulation and cellular apoptosis [44].

Collectively, these findings suggest that β-Acetoxyisovalerylalkannin exerts anti-inflammatory and anti-proliferative effects by regulating multiple metabolic pathways and maintaining metabolic homeostasis in HaCaT cells. Although our study provides compelling in vitro evidence demonstrating that β-Acetoxyisovalerylalkannin suppresses inflammation and metabolic dysregulation in HaCaT cells via the MAPK/STAT3 pathway, the lack of in vivo validation remains a limitation. Nevertheless, our integrated network pharmacology and untargeted metabolomics approach identified high-confidence targets (e.g., STAT3, GSH metabolism) associated with inflammatory skin diseases such as psoriasis, supporting the translational potential of β-Acetoxyisovalerylalkannin. Future studies should prioritize in vivo validation using animal models (e.g., IMQ-induced psoriasiform dermatitis in mice) to confirm these mechanisms in a physiological context.

## 4. Materials and Methods

### 4.1. Drugs and Reagents

#### 4.1.1. Cells

The HaCaT cell line was obtained from Cellverse Co., Ltd. (Shanghai, China) and cultured in Dulbecco’s Modified Eagle Medium (DMEM) enriched with 10% fetal bovine serum (FBS) and a 1% penicillin–streptomycin antibiotic solution. These cells were incubated at 37 °C in a humidified atmosphere containing 5% carbon dioxide, with fresh growth medium replenished every 24 h to maintain optimal conditions.

#### 4.1.2. Reagents

β-Acetoxyisovalerylalkannin (LOT: T5S2350, purity 99.69%) was purchased from Alfabiotech (Chengdu, China). Lipopolysaccharide (LPS) (LOT: 233726), interleukin 22 (IL-22) (LOT: 234237), and interleukin 17A (IL-17A) (LOT: 234759) were purchased from TargetMol Chemicals Inc. (Wellesley Hills, MA, USA).

### 4.2. Methods

#### 4.2.1. Network Pharmacology and Molecular Docking

The network pharmacology analysis was initiated by screening potential targets using keywords including “skin inflammatory diseases” and “β-Acetoxyisovalerylalkannin” across GeneCards (https://www.genecards.org, accessed on 7 January 2025), OMIM (https://www.omim.org/, accessed on 7 January 2025), and PubChem (https://pubchem.ncbi.nlm.nih.gov/, accessed on 7 January 2025). Target prediction was subsequently performed using SwissTargetPrediction (http://swisstargetprediction.ch/, accessed on 7 January 2025) with stringent thresholds (BATMAN score > 25, SwissTargetPrediction probability > 0). The overlapping targets between compounds and disease-related genes were identified using Venny 2.1.0 software, followed by prioritizing key candidate genes. Protein–protein interaction networks of the intersecting targets for both inflammatory skin diseases and β-Acetoxyisovalerylalkannin were analyzed in the STRING database (Homo sapiens, interaction score ≥ 0.40) and visualized using Cytoscape 3.10.0. Comprehensive functional enrichment analysis was conducted through Metascape (https://metascape.org/, accessed on 7 January 2025), including Gene Ontology (GO) and Kyoto Encyclopedia of Genes and Genomes (KEGG) pathway analyses. Protein structures were retrieved from the PDB database (https://www.rcsb.org/, accessed on 7 January 2025) with the following criteria: “Homo sapiens” and a refinement resolution < 3.0. Molecular docking simulations were performed using AutoDockTools 1.5.6 software. The optimal molecular models based on binding energy were visualized using PyMOL 3.0.4.

#### 4.2.2. Preparation of β-Acetoxyisovalerylalkannin Stock Solution

β-Acetoxyisovalerylalkannin was dissolved in dimethyl sulfoxide (DMSO) to create a 40 mmol·L^−1^ solution, which was subsequently aliquoted and refrigerated at −20 °C under light shielding for future use.

#### 4.2.3. Cell Viability Assay (CCK-8 Method)

HaCaT cells in the logarithmic growth phase were seeded in 96-well plates at a density of 8000 cells per well. Once the cells reached 60–70% confluence, β-Acetoxyisovalerylalkannin concentrations of 1.0, 2.0, 3.0, 4.0, 5.0, 7.5, 10, and 15.0 μmol·L^−1^ were added for 24 and 48 h. After the treatment period, each well received 100 μL of CCK-8 solution, followed by a 30 min incubation period at 37 °C. Optical density readings were taken at 450 nm using a microplate reader (Thermo Fisher, Waltham, MA, USA). Cell viability percentages were determined using the following formula: cell viability (%) = [(OD_Experimental group_ − OD_blank group_)/(OD_Control group_ − OD_blank group_)] × 100%. This calculation provided a quantitative assessment of cellular metabolic activity relative to untreated controls.

#### 4.2.4. Clone Formation Assay for Assessing Cell Proliferation

HaCaT cells that were growing exponentially were plated in six-well culture dishes at a concentration of 600 cells per well. The experimental groups received β-Acetoxyisovalerylalkannin treatment at three different concentrations (2, 4, and 8 μmol·L^−1^), with each dosage group being tested in triplicate. After 48 h of treatment, the colonies were fixed with 4% paraformaldehyde for 30 min and stained with 0.2% crystal violet for 30 min at room temperature. The number of colonies formed was recorded.

#### 4.2.5. Scratch Assay for Evaluating Cell Migration

HaCaT cells, in their log-phase of growth, were sown across six-well plates with a density of 3.5 × 10^5^ cells per well and grouped according to the protocol described in Section 4.2.4, before being cultured until they achieved about 90% confluence. We scratched the bottom of each well with the beak of a pipette, treating them with varying levels of β-Acetoxyisovalerylalkannin. We monitored scratch healing at intervals of 0, 24, and 48 h. We then employed ImageJ software (version 1.54d) to calculate the cell migration rate, using the following formula: cell migration rate (%) = (Initial scratch area *−* scratch area at 24/48 h)/Initial scratch area × 100%.

#### 4.2.6. Flow Cytometry

HaCaT cells were seeded in six-well plates at a density of 3.5 × 10^5^ cells/well and grouped according to the protocol described in Section 4.2.4. Each group was treated with varying concentrations of drug-containing medium for 48 h. Once the cells had finished incubating, the medium was carefully gathered, and the cells were detached using EDTA-free trypsin, a process known as trypsinization. Following centrifugation at 500× *g* for 5 min at 4 °C, the cell pellet was resuspended in ice-cold 1× Binding Buffer. Subsequently, 100 μL of the cell suspension was aliquoted into a flow cytometry tube, followed by the sequential addition of 5 μL of Annexin V-FITC and 5 μL of propidium iodide (PI), with gentle mixing after each reagent. The concoction was mixed delicately to ensure everything was well blended, then left to incubate in the dark, at room temperature, for 8–10 min. Post-incubation, 400 μL of pre-cooled 1× Binding Buffer was poured in, and the samples were promptly assessed using a flow cytometer (BD Biosciences, Franklin Lakes, NJ, USA) within 1 h.

#### 4.2.7. Immunofluorescence Detection of Apoptosis

HaCaT cells were plated in 12-well culture dishes at a concentration of 1 × 10^5^ cells per well and subjected to the treatment protocols outlined in Section 4.2.4. The cells were incubated with predetermined doses of β-Acetoxyisovalerylalkannin for 48 h while being maintained in complete growth medium. After the exposure period, researchers carefully removed the culture medium and performed two PBS washes to gently rinse the cell monolayers. For viability assessment, cells were incubated with 2 μmol·L^−1^ of Calcein-AM for 30 min at 37 °C in a humidified 5% CO_2_ atmosphere. After PBS washing, the PI working solution was added and incubated for 15 min at room temperature under light-protected conditions. Following two additional PBS washes, cells were immediately covered with antifade mounting medium and visualized using an inverted fluorescence microscope (Leica Microsystems CMS GmbH, Wetzlar, Germany).

#### 4.2.8. Determination of HaCaT Cell-Associated Inflammatory Factors via RT-qPCR

HaCaT cells were seeded in six-well plates at 5 × 10^5^ cells/well and divided into groups: the control group, the IL-17A model group, the LPS model group, and the β-Acetoxyisovalerylalkannin-treated groups (low and high doses: 2 μmol·L^−1^ and 4 μmol·L^−1^). To establish the inflammatory model, cells were exposed to a combination of 25 ng·mL^−1^ of IL-17A and 500 ng·mL^−1^ of LPS for a 48 h period. After treatment, total RNA was extracted from all experimental groups using the FreeZol Reagent kit, followed by conversion to complementary DNA (cDNA) through reverse transcription. Gene expression analysis was conducted via quantitative real-time PCR (qRT-PCR) employing SYBR Green master mix on a QuantStudio Real-Time Fluorescence Quantitative PCR Instrument (Thermo Fisher, Waltham, MA, USA). Target gene mRNA levels were quantified relative to the housekeeping gene *β-actin*, with expression values determined through the 2^−ΔΔCt^ calculation method. The complete set of primer sequences utilized in the amplification process is shown in Table 2.

#### 4.2.9. Western Blot Detection of MAPK/STAT3 Signaling Pathway Protein Expression

HaCaT keratinocytes were seeded in 6-well plates at a density of 3.5 × 10^5^ cells per well and randomly assigned to the following experimental groups: untreated control, IL-22-induced model (100 ng·mL^−1^ IL-22), and β-Acetoxyisovalerylalkannin treatment groups (2, 4, and 8 μmol·L^−1^) co-treated with IL-22. After 48 h of stimulation, the cells were lysed using RIPA buffer containing protease and phosphatase inhibitors (1 mM PMSF and a 1× inhibitor cocktail). Protein levels were quantified via a BCA assay, following the manufacturer’s instructions. Equal amounts of protein (30 μg per lane) were heat-denatured in 5× loading buffer at 95 °C for 10 min before being separated on 10% SDS–polyacrylamide gels. Electrophoresis was carried out under standard conditions—initially at 45 V for 45 min, followed by 120 V for 55 min. Proteins were transferred onto 0.45 μm of PVDF membranes at 500 mA for 60 min in ice-cold transfer buffer. Membranes were blocked with 5% (*w*/*v*) non-fat dry milk in TBST for 1 h at room temperature, then incubated overnight at 4 °C with primary antibodies against Phospho-STAT3 (Y705) (1:2000, Cat# GB150001), STAT3 (1:1500, Cat# GB12176), Phospho-ERK1/2 (T202/Y204) (1:800, Cat# GB11004), ERK1/2 (1:2000, Cat#GB11560), Phospho-p38 (1:2000, Cat#GB153380), p38 (1:2000, Cat#GB154685), and β-actin (1:2000, Cat#GB15003). Following three 10 min TBST rinses, the membranes were treated with HRP-linked secondary antibodies (diluted 1:5000) for one hour at ambient temperature. Protein bands were visualized using an enhanced chemiluminescence (ECL) substrate and captured with a chemiluminescence imaging system (VILBERLOURMAT, Collégien, France). Quantitative analysis of band intensities was performed using Image Lab software (version 6.1, Bio-Rad, Hercules, CA, USA), with values normalized against their respective loading controls.

#### 4.2.10. Non-Targeted Metabolomics

HaCaT cells were planted in six-well dishes at a concentration of 5 × 10^5^ cells per dish and then split into three categories: the control set, the IL-17A model group (25 ng·mL^−1^), and the β-Acetoxyisovalerylalkannin group (4 μmol·L^−1^). The model and experimental groups were treated with the corresponding drug-containing medium and incubated for 48 h. After treatment, cells were lysed with 80% pre-cooled acetonitrile and centrifuged at 10 min, 4 °C, and 12,000× *g*. The supernatant was dried under nitrogen and reconstituted in 50% acetonitrile, followed by centrifugation at 4 °C for 10 min. The supernatant was then collected into an injection vial for analysis. The UPLC-Q-TOF/MS (Xevo G2-XSQ-TOF Quadrupole Time-of-Flight Mass Spectrometer; Waters, Milford, MA, USA) detection involved specific chromatographic conditions. Mobile phase A was a buffered aqueous solution with a trace of 0.1% formic acid, while mobile phase B was composed of pure acetonitrile. We utilized a gradual gradient elution scheme that began at 2% of phase B at 0–0.5 min, then increased to 7% over the next 15 min, increased further to 40% between 15 and 22 min, increased again to 67% from 22 to 25 min, and finally reached 100% at 25 min, before decreasing to the starting point of 2% by the 40 min mark. The flow rate was set to a consistent 0.3 mL·min^−1^, and a 5 μL injection was used. The column was kept cool at 35 °C throughout the process. In terms of mass spectrometry, we employed electrospray ionization in both positive and negative ion modes, with a scan spectrum that spanned from 50 to 1200 Daltons and took one second per scan. The cone voltage was 40 V, and the ionization was 120 °C, with the collision energy varying from a modest 10 eV to a notable 45 eV. For a fail-safe quality check, we prepared a QC sample by merging the equivalent parts of all experimental samples and ran it alongside the others to ensure uniform quality standards.

#### 4.2.11. Data Analysis and Processing

The raw data were processed using Progenesis QI metabolomics software (version 2.0, Waters) for feature extraction, alignment, and normalization. Total ion chromatograms (TICs) were generated to evaluate data quality. Metabolite identification was performed by matching mass spectra against the Human Metabolome Database (HMDB, https://www.hmdb.ca/, accessed on 30 January 2025). Multivariate statistical analyses, including principal component analysis (PCA) and orthogonal partial least squares–discriminant analysis (OPLS-DA), were conducted using EZinfo 3.0 software (version 3.0, Waters, USA). Metabolites with Variable Importance in Projection (VIP) values > 1 and statistical significance (*p* < 0.05) determined through one-way ANOVA were selected for further analysis. The relevant metabolic pathways were explored using MetaboAnalyst 5.0 software (https://www.metaboanalyst.ca/, accessed on 30 January 2025). Data are presented as the mean ± standard error of the mean (S.E.M). Statistical data were analyzed and visualized using GraphPad Prism 9.5 software (USA). Statistical analysis was conducted using an independent-samples *t*-test for comparisons between two groups and one-way ANOVA for comparisons between multiple groups. A *p*-value of <0.05 was considered statistically significant.

## 5. Conclusions

β-Acetoxyisovalerylalkannin potently suppresses the excessive proliferation of HaCaT cells, reduces the levels of inflammation-related molecules, and modulates the MAPK/STAT3 signaling pathway to exert anti-inflammatory and anti-proliferative effects in vitro. Additionally, it may influence the process of skin inflammation through metabolic pathways such as arginine and proline metabolism, as well as glutathione metabolism. This study provides a theoretical foundation for understanding the anti-inflammatory and anti-proliferative effects of β-Acetoxyisovalerylalkannin on HaCaT cells, suggesting its potential use as a therapeutic agent for treating inflammatory skin diseases. However, this study is limited to in vitro experiments, and further in vivo studies are required to fully evaluate the efficacy, safety, and potential long-term adverse effects of β-Acetoxyisovalerylalkannin in animal models. These future studies will be essential for supporting the development of novel treatments for dermatitis.

## Figures and Tables

**Table 1:** Endogenous differential metabolites in β-Acetoxyisovalerylalkannin and IL-17A groups (top 20).

Serial Number	Retention Time (min)	Mass-to-Charge Ratio *m*/*z*	Metabolite Name	Molecular Formula	One-Way ANOVA (*p*)	β-Acetoxyisovalerylalkannin Treatment
1	2.004383	415.2105	Armillarin	C_24_H_30_O_6_	0.000243	↑
2	1.65725	792.5764	PG(18:1(9*Z*)/18:1(9*Z*))	C_42_H_79_O_10_P	0.00014	↑
3	9.9014	182.0808	Coumarinic acid	C_9_H_8_O_3_	0.011782	↑
4	6.342983	116.107	(*Z*)-4-Hexenal	C_6_H_10_O	3.21 × 10^−7^	↓
5	1.969567	359.3147	MG(18:0/0:0/0:0)	C_21_H_42_O_4_	3.56 × 10^−5^	↑
6	10.36602	608.0886	Uridine diphosphate-*N*-acetylglucosamine	C_17_H_27_N_3_O_17_P_2_	0.005755	↑
7	10.73917	130.0499	Pyroglutamic acid	C_5_H_7_NO_3_	7.80 × 10^−6^	↑
8	2.833333	138.1274	1,2,4-Tris(methylene)cyclohexane	C_9_H_12_	0.000432	↓
9	9.24125	166.0859	Cinnamic acid	C_9_H_8_O_2_	0.000894	↑
10	10.33287	308.0913	1-[4,9-Dihydro-2-(methylthio)-1,3-thiazino[6,5-*b*]indol-4-yl]-2-propanone	C_14_H_14_N_2_OS_2_	0.005284	↑
11	1.787267	391.2838	12-Ketodeoxycholic acid	C_24_H_38_O_4_	0.001827	↑
12	14.50635	161.128	L-2-Amino-3-methylenehexanoic acid	C_7_H_13_NO_2_	0.014649	↑
13	14.3875	130.0862	L-*trans*-4-Methyl-2-pyrrolidinecarboxylic acid	C_6_H_11_NO_2_	1.65 × 10^−5^	↑
14	2.548467	675.5417	SM(d18:1/14:0)	C_37_H_75_N_2_O_6_P	0.017606	↑
15	1.969567	331.2832	MG(0:0/16:0/0:0)	C_19_H_38_O_4_	0.00129	↑
16	10.89645	508.0031	dGTP	C_10_H_16_N_5_O_13_P_3_	0.000442	↑
17	10.33287	615.1723	Safflomin C	C_30_H_30_O_14_	0.000167	↑
18	10.36602	148.0603	L-Glutamic acid	C_5_H_9_NO_4_	3.72 × 10^−7^	↑
19	10.36602	130.0498	1-Pyrroline-4-hydroxy-2-carboxylate	C_5_H_7_NO_3_	0.000189	↑
20	10.77292	86.09667	Piperidine	C_5_H_11_N	4.85 × 10^−6^	↓

“↑” indicates an upward adjustment and “↓” indicates a downward adjustment.

**Table 2:** Primer sequences.

Primers	Forward Sequence (5′-3′)	Reverse Sequence (5′-3′)
*IL-17A*	TCAGCGTGTCCAAACACTGAG	CGCCAAGGGAGTTAAAGACTT
*CXCL1*	CTGGGATTCACCTCAAGAACATC	CAGGGTCAAGGCAAGCCTC
*CXCL2*	ATGCCTCACTCGTACCCAG	TTTCCACCCCAATTTGGCTCA
*CXCL8*	ACTGAGAGTGATTGAGAGTGGAC	AACCCTCTGCACCCAGTTTTC
*CCL20*	ACTGTTGCCTCTCGTACATACA	GAGGAGGTTCACAGCCCTTTT
*IFN-γ*	GTGATGGCTGAACTGTCGCC	CTGGGATGCTCTTCGACCTC
*MCP-1*	CAGCCAGATGCAATCAATGCC	TGGAATCCTGAACCCACT
*NF-κB*	ACACCCACAAACCAACTCTGG	TGCTGAACACTGGAGGAAGTC
*TNF-α*	TCCAGTGTGTCCTTCCGAAGT	TGCCTCCGCCAGAACTGTA
*β-actin*	GGCTGTATTCCCCTCCATCG	CCAGTTGGTAACAATGCCATGT

## Data Availability

Data presented in this study is contained within the article. Further inquiries can be directed to the corresponding author.
